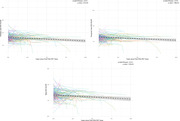# Prevalence of hypometabolism in AD‐related regions in real‐world FDG‐PET of the brain

**DOI:** 10.1002/alz.093991

**Published:** 2025-01-09

**Authors:** Trent Schwartz, W Hudson Robb, Steven Bishay, T. Bryan Jackson, Angela L. Jefferson, Bennett A. Landman, David Samuel Smith, Timothy J. Hohman, Mary Ellen I. Koran

**Affiliations:** ^1^ Vanderbilt Memory and Alzheimer's Center, Vanderbilt University Medical Center, Nashville, TN USA; ^2^ Vanderbilt Memory & Alzheimer's Center, Vanderbilt University Medical Center, Nashville, TN USA; ^3^ Vanderbilt University School of Medicine, Nashville, TN USA; ^4^ Department of Biomedical Engineering, Vanderbilt University, Nashville, TN USA; ^5^ Vanderbilt University Institute of Imaging Sciences, Vanderbilt University Medical Center, Nashville, TN USA; ^6^ Vanderbilt Memory & Alzheimer’s Center, Vanderbilt University Medical Center, Nashville, TN USA

## Abstract

**Background:**

Standard of care for many cancer workups includes whole‐body FDG PET/CT before, during, and after therapy. At Vanderbilt, these scans include the brain for every patient (>20,000 patients). Brain FDG PET is a validated assessment of neuronal health. We will use these standard of care FDG PET images as incidental screening opportunities to evaluate neuronal health to leverage in future electronic health record (EHR) research.

**Methods:**

Demographic data and FDG images were extracted from Vanderbilt’s EHR for the most recently imaged 1000 patients. Brain images were extracted, quality control was performed, and SUVR for an established AD meta‐region of interest (ROI) was calculated (Landau, et.al, 2011) as were SUVR for AD‐related ROIs including parietal and temporal lobes. We evaluated the prevalence of a positive AD metaROI (SUVR <=1.21) at staging and on post‐treatment images as well as change in ROI SUVR over time using a linear mixed effects model with subject specific slopes and intercepts, covarying for baseline age and sex.

**Results:**

396 patients >=65 years old were included in this analysis. Most common indications for imaging included lymphoma, melanoma, and head and neck and lung cancers. 159 have >=2 FDG, with an average follow up time of 338 days between staging and follow‐up scans. 272 patients (68.6%) had a positive AD metaROI (<=1.21) at their initial staging PET, which increased to 296 patients (74%) on follow up. AD metaROI SUVR decreased over time in these patients (β(ΔSUVR/year)=‐0.013 and p=1.63x10‐4), and in the parietal (β=‐0.014/year and p=2.57x10‐5) and temporal (β(ΔSUVR/year)=‐0.010 and p=1.68x10‐4) lobes (Figure 1).

**Conclusion:**

Incidental prevalence of a positive AD metaROI on FDG is high compared to expected prevalence of a clinical diagnosis of AD of ∼10%. Thus published cut‐points may not be applicable to PET of the brain captured during whole body imaging; different cut‐points may be required. FDG in AD‐related regions significantly decreased over time, consistent with expected age‐related changes. Incidental evaluation of the cerebral metabolism may hold important information on brain health. Evaluation of the entire cohort (n>20,000) and comparison to age‐matched controls is ongoing.